# Non-canonical *WOX11*-mediated root branching contributes to plasticity in *Arabidopsis* root system architecture

**DOI:** 10.1242/dev.152132

**Published:** 2017-09-01

**Authors:** Lihong Sheng, Xiaomei Hu, Yujuan Du, Guifang Zhang, Hai Huang, Ben Scheres, Lin Xu

**Affiliations:** 1 National Key Laboratory of Plant Molecular Genetics, CAS Center for Excellence in Molecular Plant Sciences, Institute of Plant Physiology and Ecology, Shanghai Institutes for Biological Sciences, Chinese Academy of Sciences, 300 Fenglin Road, Shanghai 200032, China; 2 University of Chinese Academy of Sciences, 19A Yuquan Road, Beijing 100049, China; 3 Plant Developmental Biology Group, Wageningen University Research, Droevendaalsesteeg 1, 6708 PB Wageningen, The Netherlands

**Keywords:** Adventitious root, Lateral root, *LBD16*, *WOX11*, *Arabidopsis*, Root system

## Abstract

Lateral roots (LRs), which originate from the growing root, and adventitious roots (ARs), which are formed from non-root organs, are the main contributors to the post-embryonic root system in *Arabidopsis*. However, our knowledge of how formation of the root system is altered in response to diverse inductive cues is limited. Here, we show that *WOX11* contributes to root system plasticity. When seedlings are grown vertically on medium, *WOX11* is not expressed in LR founder cells. During AR initiation, *WOX11* is expressed in AR founder cells and activates *LBD16*. *LBD16* also functions in LR formation and is activated in that context by *ARF7*/*19* and not by *WOX11*. This indicates that divergent initial processes that lead to ARs and LRs may converge on a similar mechanism for primordium development. Furthermore, we demonstrated that when plants are grown in soil or upon wounding on medium, the primary root is able to produce both *WOX11*-mediated and non-*WOX11*-mediated roots. The discovery of *WOX11*-mediated root-derived roots reveals a previously uncharacterized pathway that confers plasticity during the generation of root system architecture in response to different inductive cues.

## Abstract (precis)

**Summary:** Root system development can respond flexibly to developmental and environmental cues by utilizing *WOX11*-mediated and non-*WOX11*-mediated pathways, which converge on a common mechanism for primordium development involving *LBD16*.

## INTRODUCTION

The plant root system is a complex result of iterative developmental processes, including formation of the primary root during embryogenesis and production of new root primordia after germination, including lateral roots (LRs) and adventitious roots (ARs) (Lavenus et al., 2013; Atkinson et al., 2014; Bellini et al., 2014). In *Arabidopsis thaliana*, LRs are initiated from the xylem-pole pericycle cells of a growing root in a developmentally and periodically organized pattern (Peret et al., 2009; Moreno-Risueno et al., 2010; Lavenus et al., 2013; Van Norman et al., 2014; Xuan et al., 2015, 2016), whereas ARs are described as being formed in non-root organs such as the hypocotyl and detached stem and leaf explants primarily in response to environmental cues, such as wounding or stress (Bellini et al., 2014; Xu and Huang, 2014; Rellan-Alvarez et al., 2016). However, how the root system is plastically formed in response to diverse inductive cues when plants are grown in different conditions is barely understood.

The cellular and molecular processes key to LR formation from the primary root grown vertically on medium have been extensively investigated (de Smet, 2012; Laskowski, 2013; Vermeer and Geldner, 2015). Generally, an oscillatorily root cap-derived flux of auxin signaling is thought to prime the LR founder cells (Moreno-Risueno et al., 2010; Xuan et al., 2015, 2016) which activates AUXIN RESPONSE FACTOR7 (ARF7) and ARF19 (Okushima et al., 2007) transcription factors. In turn, ARF7 and ARF19 upregulate LATERAL ORGAN BOUNDARIES DOMAIN (LBD) genes for the initiation of a lateral root primordium (LRP) (Okushima et al., 2007; Lee et al., 2009; Goh et al., 2012).

We previously reported that *WUSCHEL-RELATED HOMEOBOX11* (*WOX11*) is required for *de novo* regeneration of ARs from leaf explants (Liu et al., 2014), raising the possibility that *WOX11* is involved in AR initiation. In the current study, we show that *WOX11* is not involved in LR initiation when plants are vertically grown on medium, and that the primary root can produce both *WOX11*-mediated roots and *ARF7*/*19*-mediated LRs in response to different inductive cues when plants are grown in soil or upon wounding on medium. The different root system architecture of plants grown in different conditions suggests flexibility of rooting pathways in response to developmental and environmental cues.

## RESULTS

### *WOX11* does not contribute to LR initiation when plants are vertically grown on medium

**Fig. 1.**
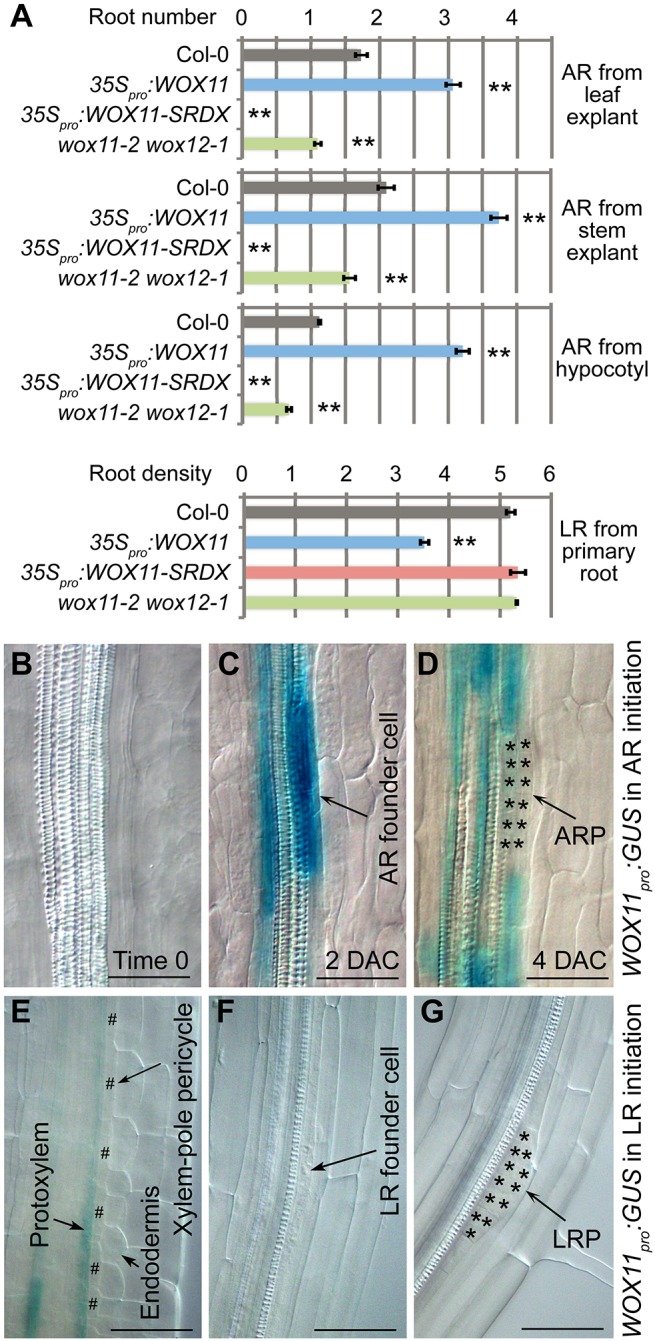
**Analysis of *WOX11* in AR and LR formation on medium.** (A) Statistical analysis of root number per leaf explant at 12 DAC, per stem explant at 12 DAC, per hypocotyl and per 1-cm primary root. LR analysis includes emerged LRs together with LRP observed under DIC microscopy. Error bars represent s.e.m. from three biological repeats. *n*=30 (leaf explant, stem explant and hypocotyl) or *n*=10 (primary root) in each repeat. **P<0.01 (two-sample Student's *t*-test compared with Col-0). (B-D) GUS staining of *WOX11_pro_:GUS* from leaf explant at time 0 (B), 2 DAC (C) and 4 DAC (D). Note that the GUS signal was detected specifically in the AR founder cells (C) (Liu et al., 2014). We observed more than 20 leaf explants from two independent lines and all of them showed GUS staining in AR founder cells. (E-G) GUS staining of *WOX11_pro_:GUS* in the primary root. Note that GUS signal was detected in the protoxylem at the meristematic region (E) as previously reported (Liu et al., 2014), but was not detected in xylem-pole pericycle cells (E), LR founder cells (F) or LRP (G). We observed more than 20 seedlings from two independent lines and none of them showed GUS staining in LR founder cells. Also see Fig. S4D-F. Leaf explants (A-D) and stem explants (A) were detached from 12- and 24-day-old seedlings grown on 1/2 MS medium, respectively. The detached explants were then cultured on B5 medium without added hormones (Chen et al., 2014). Hypocotyls (A) were analyzed using 14-day-old seedlings vertically grown on 1/2 MS medium. Primary and lateral roots were analyzed using 9-day-old (A) or 7-day-old (E-G) seedlings vertically grown on 1/2 MS medium. Asterisks in D and G indicate root primordium cells, and hash symbols in E indicate xylem-pole pericycle. Scale bars: 50 μm.

To analyze the role of *WOX11*, we exploited adventitious rooting systems in *Arabidopsis* from leaf explants (Fig. 1A-D; Fig. S1A,E,I), stem explants (Fig. 1A; Fig. S1B,F,J) and hypocotyls (Fig. 1A; Fig. S1C,G,K). Detached leaf and stem explants generate ARs within 8 and 6 days after culture (DAC) on B5 medium, respectively (Chen et al., 2014). We also observed lateral rooting from the primary root grown vertically on medium (Fig. 1A,E-G; Fig. S1D,H,L). All plants and explants were grown and cultured *in vitro* on medium.

We examined the role of *WOX11* in these rooting systems using two transgenic constructs, *35S_pro_:WOX11* and *35S_pro_:WOX11-SRDX*, to enhance and block *WOX11* activity, respectively (Liu et al., 2014). Compared with wild-type Columbia-0 (Col-0) (Fig. 1A; Fig. S1A-C), AR formation was greatly enhanced in leaf explants, stem explants and hypocotyls of *35S_pro_:WOX11* plants (Fig. 1A; Fig. S1E-G), whereas AR production was severely inhibited in *35S_pro_:WOX11-SRDX* plants (Fig. 1A; Fig. S1I-K). However, compared with Col-0, no defects in LR density in *35S_pro_:WOX11-SRDX* or enhanced LR density in *35S_pro_:WOX11* were observed (Liu et al., 2014) (Fig. 1A; Fig. S1D,H,L), although *35S_pro_:WOX11-SRDX* had a shorter primary root than Col-0 (Fig. S2). Instead, LR density in *35S_pro_:WOX11* plants was reduced (Fig. 1A; Fig. S1H). This response might be caused by a feedback process due to enhanced adventitious rooting of hypocotyls induced by *WOX11* overexpression (Fig. 1A; Fig. S1G).

*WOX11* and *WOX12* are partially redundant genes (Liu et al., 2014). To understand further the roles of *WOX11* and *WOX12* in adventitious rooting, we analyzed the phenotype of a *wox11-2 wox12-1* double mutant. Adventitious rooting from leaf explants, stem explants and hypocotyls were defective in *wox11-2 wox12-1* compared with Col-0 (Fig. 1A), although the defects were milder than those in *35S_pro_:WOX11-SRDX* plants. In contrast to the defective adventitious rooting, lateral root density remained normal in the *wox11-2 wox12-1* mutant (Fig. 1A).

Next, we compared the expression pattern of *WOX11* during AR and LR formation using *WOX11_pro_:GUS* transgenic lines (Liu et al., 2014). *WOX11* promoter activity was confirmed by introducing *WOX11_pro_:WOX11* into the *wox11-2 wox12-1* background, which complemented the adventitious rooting defect (Fig. S3). *WOX11* was induced in all examined AR-competent tissues during AR formation (Fig. S4A-C). A detailed analysis using leaf explants showed that *WOX11* was initially induced in AR founder cells within 2 DAC (Fig. 1B,C; Fig. S4J,K) (Liu et al., 2014). Expression of *WOX11* then gradually decreased in the adventitious root primordium (ARP) as it developed (Fig. 1D; Fig. S4L) (Liu et al., 2014).

In contrast, GUS signal was not detectable during LR formation (Fig. S4D-F). More specifically, *WOX11* expression was absent in xylem-pole pericycle cells (Fig. 1E; Fig. S4E,F), LR founder cells (Fig. 1F; Fig. S4E,F) and LRP (Fig. 1G; Fig. S4E,F). Instead, expression was detected in protoxylem cells in the primary root meristematic region (Fig. 1E; Fig. S4E) (Liu et al., 2014). In addition, quantitative reverse transcription-polymerase chain reaction (qRT-PCR) analysis confirmed the expression of *WOX11* during AR but not LR formation (Fig. S4G-I). Therefore, LR formation from the primary root grown vertically on medium is controlled by the non-*WOX11*-mediated pathway.

In addition, we performed experiments in different growth conditions and confirmed that *WOX11* was also involved in light control of adventitious rooting from hypocotyls (Sorin et al., 2005; Gutierrez et al., 2009) (Fig. S5). We tested *WOX11* expression patterns in leaf explants and primary roots by culturing them on different medium, and the results showed that change of the medium had little effect on *WOX11* expression (Fig. S6).

In summary, *WOX11* was specifically expressed in diverse adventitious rooting systems but not in LR formation from the primary root grown vertically on medium. Therefore, when plants are vertically grown on medium, lateral rooting follows the non-*WOX11*-mediated pathway, which requires *ARF7*/*19*.

### The primary root is able to produce both *WOX11*-mediated roots and non-*WOX11*-mediated roots when plants are grown in soil or upon wounding

According to traditional views, *Arabidopsis* LRs derive from xylem-pole pericycle cells of an existing root and the initiation of all LRs is usually considered to be dependent on identical processes. Here, we re-examined ‘lateral root’ formation by analyzing whether *WOX11*-mediated roots can develop from the primary root.

**Fig. 2.**
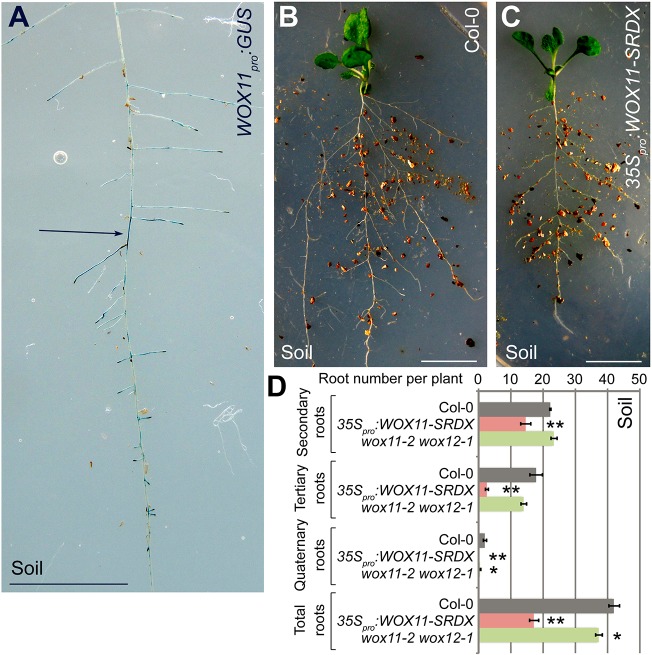
***WOX11*-mediated rooting contributes to the root system derived from the primary root in soil.** (A) GUS staining of an 18-day-old *WOX11_pro_:GUS* plant grown in soil. The GUS signal was observed in many regions of the root (arrow). Seedlings were carefully moved out of the pot, together with the soil that was adhered to the root, for GUS staining. After GUS staining, the soil was washed away before observation. (B,C) The primary root and its root system from 18-day-old Col-0 (B) and *35S_pro_:WOX11-SRDX* (C) grown in soil. (D) Statistical analysis of the root systems of 18-day-old Col-0, *35S_pro_:WOX11-SRDX* and *wox11-2 wox12-1* plants grown in soil. Error bars represent s.e.m. from three biological repeats. *n*=10 plants for each repeat. *P<0.05, **P<0.01 (two-sample Student's *t*-test compared with Col-0). Also see Fig. S8. Scale bars: 1 cm.

LR initiation from wild-type seedlings grown vertically on half-strength Murashige and Skoog (1/2 MS) medium or some other media usually did not display a *WOX11* signal and followed the non-*WOX11*-mediated rooting pathway (Fig. 1E-G; Fig. S4D-F; Fig. S6). However, in natural conditions, plants are grown in soil. Therefore, we tested whether the root system grown in soil conditions is able to produce *WOX11*-mediated roots. Strikingly, in *WOX11_pro_:GUS* plants grown in soil, GUS signal was detected in many regions of the root system (Fig. 2A; Fig. S7). In addition, secondary, tertiary and quaternary root numbers were all reduced in the root system of *35S_pro_:WOX11-SRDX* seedlings grown in soil compared with those of Col-0 (Fig. 2B-D). Although *35S_pro_:WOX11-SRDX* had a shorter primary root than Col-0 (Fig. S2), the density of the secondary roots derived from the primary roots was also reduced in *35S_pro_:WOX11-SRDX* seedlings grown in soil compared with those of Col-0 (Fig. S8). In *wox11-2 wox12-1* seedlings grown in soil, the number of quaternary roots was reduced compared with those of Col-0 (Fig. 2D). In *35S_pro_:WOX11* seedlings grown in soil, the numbers of tertiary and quaternary roots were enhanced compared with those of Col-0 (Fig. S9). Taken together, these data suggest that in soil conditions, *WOX11*-mediated root formation does occur in the root system, which may reflect a response to environmental signals in soil.

When plants are grown in soil, they may encounter different nutrition conditions, different types of stresses, damage of roots, and interactions with microbe and insects. We investigated whether damage of the primary root is able to induce *WOX11*-mediated root formation. The primary root of 12-day-old *WOX11_pro_:GUS* seedlings vertically grown on 1/2 MS medium was cut at the midpoint to mimic damage of roots. GUS signals could be detected at the excision site within 1 day after excision (DAE) (Fig. S10), suggesting that *WOX11*-mediated root initiation might occur upon excision of the wild-type primary root tip.

**Fig. 3.**
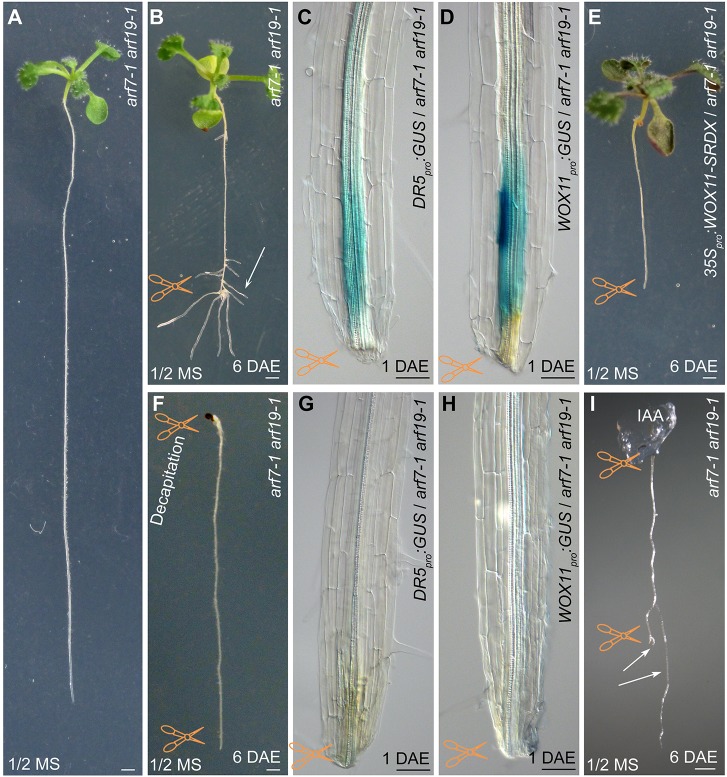
**Root formation from the *arf7-1 arf19-1* primary root upon wounding.** (A) The *arf7-1 arf19-1* mutant shows defective LR initiation. (B) Formation of roots (arrow) on wounded primary root of *arf7-1 arf19-1* at 6 DAE. (C,D) GUS staining of *DR5_pro_:GUS* (C) and *WOX11_pro_:GUS* (D) in the wounded primary root of *arf7-1 arf19-1* at 1 DAE. (E) Repression of root formation on wounded primary root of *arf7-1 arf19-1* in the *35S_pro_:WOX11-SRDX* background at 6 DAE. (F) Decapitation by removal of the above-ground tissues including the shoot and the hypocotyl resulted in loss of rooting capability on the wounded primary root of *arf7-1 arf19-1* at 6 DAE. (G,H) GUS staining of *DR5_pro_:GUS* (G) and *WOX11_pro_:GUS* (H) in the wounded primary root of *arf7-1 arf19-1* with decapitation (indicated in F) at 1 DAE. (I) Rescue of the rooting capability (arrows) by application of 1 μM indole-3-acetic acid in an agar mass to the decapitated region (indicated in F). Twelve-day-old seedlings vertically grown on 1/2 MS medium were used for analysis. Scissors indicate the cutting sites. Scale bars: 1 mm (A,B,E,F,I); 50 μm (C,D,G,H).

To eliminate interference from non-*WOX11*-mediated roots, i.e. *ARF7*/*19*-mediated LRs, we performed experiments using the *arf7-1 arf19-1* double mutant, which is defective in LR initiation when plants are grown vertically on 1/2 MS medium (Fig. 3A) (Okushima et al., 2007). The primary root of 12-day-old *arf7-1 arf19-1* seedlings vertically grown on 1/2 MS medium was cut at the midpoint to mimic damage of roots (Fig. 3A). Surprisingly, many clusters of roots were formed near the wounded region at 6 DAE (Fig. 3B). Using an auxin reporter *DR5_pro_:GUS* line, we observed that GUS staining accumulated in the wounded region at 1 DAE (Fig. 3C). In *WOX11_pro_:GUS-*marked *arf7-1 arf19-1* roots, GUS signal was detected in pericycle cells and some other vascular cells in the wounded region at 1 DAE (Fig. 3D). Moreover, introgression of *35S_pro_:WOX11-SRDX* into *arf7-1 arf19-1* resulted in defective rooting from the wounded primary root at 6 DAE (Fig. 3E). These data indicate that the roots from the wounded *arf7-1 arf19-1* primary root were formed following the *WOX11*-mediated rooting pathway rather than the *ARF7*/*19*-mediated LR pathway.

When the above-ground portion was removed from the junction between root and hypocotyl, the remaining *arf7-1 arf19-1* primary root grown vertically on 1/2 MS medium lost the ability to form roots upon wounding (Fig. 3F), and GUS staining was barely observed near the wounded region from the cut in the primary roots from both *DR5_pro_:GUS*/*arf7-1 arf19-1* and *WOX11_pro_:GUS*/*arf7-1 arf19-1* seedlings (Fig. 3G,H). The application of auxin specifically to the decapitated region at the hypocotyl-root junction of *arf7-1 arf19-1* could partially rescue the rooting defect, as the cut region of the primary root retained the ability to develop roots (Fig. 3I). These results suggest that long-distance transport of auxin from above-ground tissues (i.e. basipetal auxin flux) is required for *WOX11*-mediated root formation in the *arf7-1 arf19-1* primary root upon wounding.

**Fig. 4.**
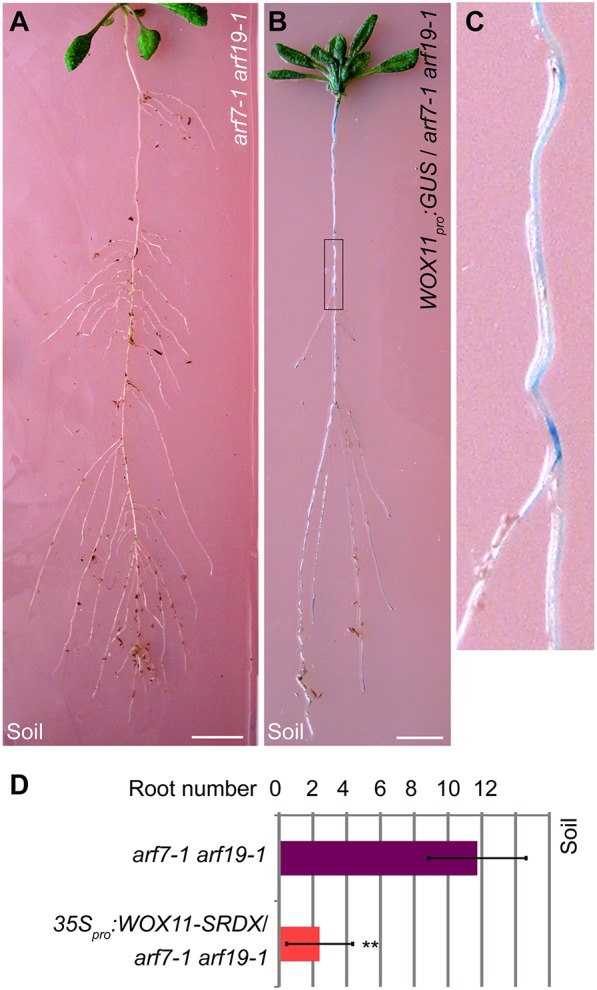
**Root formation of *arf7-1 arf19-1* in soil.** (A) The primary root and its root system from 18-day-old *arf7-1 arf19-1* grown in soil. (B) GUS staining of the primary root and its root system from 18-day-old *WOX11_pro_:GUS*/*arf7-1 arf19-1* grown in soil. (C) Magnification of the boxed region in B showing GUS signal in more detail. (D) Statistical analysis of root number from the primary root of 18-day-old *arf7-1 arf19-1* or *35S_pro_:WOX11-SRDX*/*arf7-1 arf19-1* grown in soil. Error bars represent s.e.m. from three biological repeats. *n*=10 for each repeat. **P<0.01 (two-sample Student's *t*-test compared with *arf7-1 arf19-1*). Scale bars: 1 cm (A,B).

**Fig. 5.**
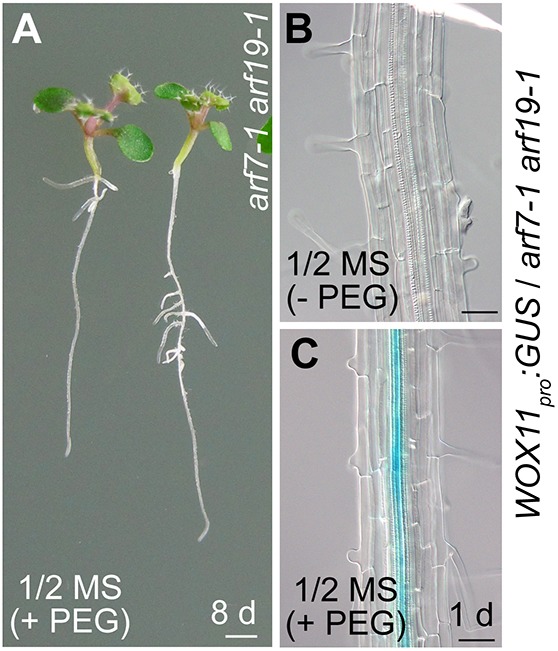
**The primary root and its root system from *arf7-1 arf19-1* under drought conditions.** (A) Root formation from the primary root and the hypocotyl of *arf7-1 arf19-1* under polyethylene glycol (PEG) treatment. (B,C) GUS staining of the primary root from *WOX11_pro_:GUS*/*arf7-1 arf19-1* before PEG treatment (−PEG; B) and after PEG treatment (+PEG; C). The plants were grown vertically on 1/2 MS medium after germination for 6 days (B) and then transferred to 1/2 MS medium containing 5% PEG to mimic the drought condition (Verslues et al., 2006) for 8 days (A) or 1 day (C). Scale bars: 1 mm (A); 50 μm (B,C).

Furthermore, many roots can be formed from the primary root of *arf7-1 arf19-1* in soil (Fig. 4A) and these roots were formed following the *WOX11*-mediated rooting pathway (Fig. 4B-D). Drought conditions may also promote root formation from the primary root of *arf7-1 arf19-1* vertically grown on 1/2 MS medium (Fig. 5A) and induce *WOX11* expression in the primary root of *arf7-1 arf19-1* (Fig. 5B,C).

Taken together, our results reveal a novel root-derived root induction process, which extends the plasticity of root system architecture.

### *WOX11*-mediated and non-*WOX11*-mediated root initiation converges on the activation of *LBD16*

**Fig. 6.**
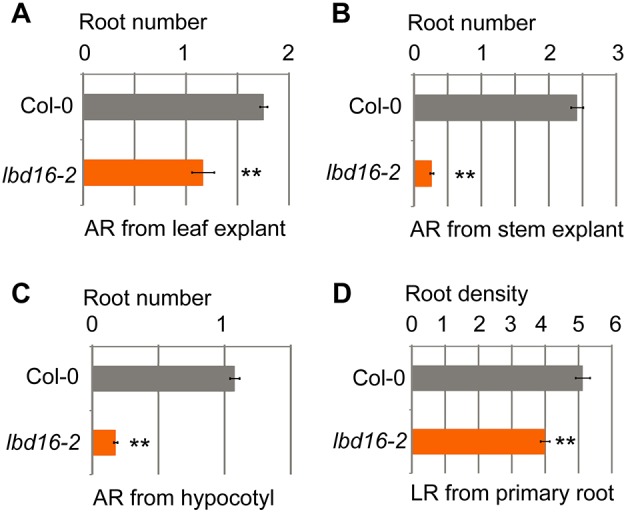
***LBD16* involvement in lateral and adventitious rooting.** (A-D) Statistical analyses of root number per leaf explant (A), per stem explant (B), per hypocotyl (C) and per 1-cm primary root (D). Error bars represent s.e.m. with three biological repeats. *n*=30 (leaf explant, stem explant and hypocotyl) or *n*=10 (primary root) in each repeat. **P<0.01 (two-sample Student's *t*-test compared with Col-0). The plant material conditions were the same as described in Fig. 1A.

Direct activation of *LBD16* by ARF7 and ARF19 is a key step during LRP initiation in the non-*WOX11*-mediated rooting pathway (Okushima et al., 2007; Lee et al., 2009; Goh et al., 2012). We observed that a *LBD16* loss-of-function mutant, *lbd16-2* (Fan et al., 2012), was defective in AR formation (Fig. 6A-C) and, to a lesser extent, in LR formation (Fig. 6D) when grown vertically on medium, suggesting that *LBD16* is involved in both *WOX11*-mediated and non-*WOX11*-mediated rooting pathways.

Genetic data showed that *WOX11* acted upstream of *LBD16* during AR formation from leaf explants (Liu et al., 2014) (Fig. S11). Here, we used several approaches to test whether WOX11 is able to regulate *LBD16* directly during AR formation from leaf explants.

**Fig. 7.**
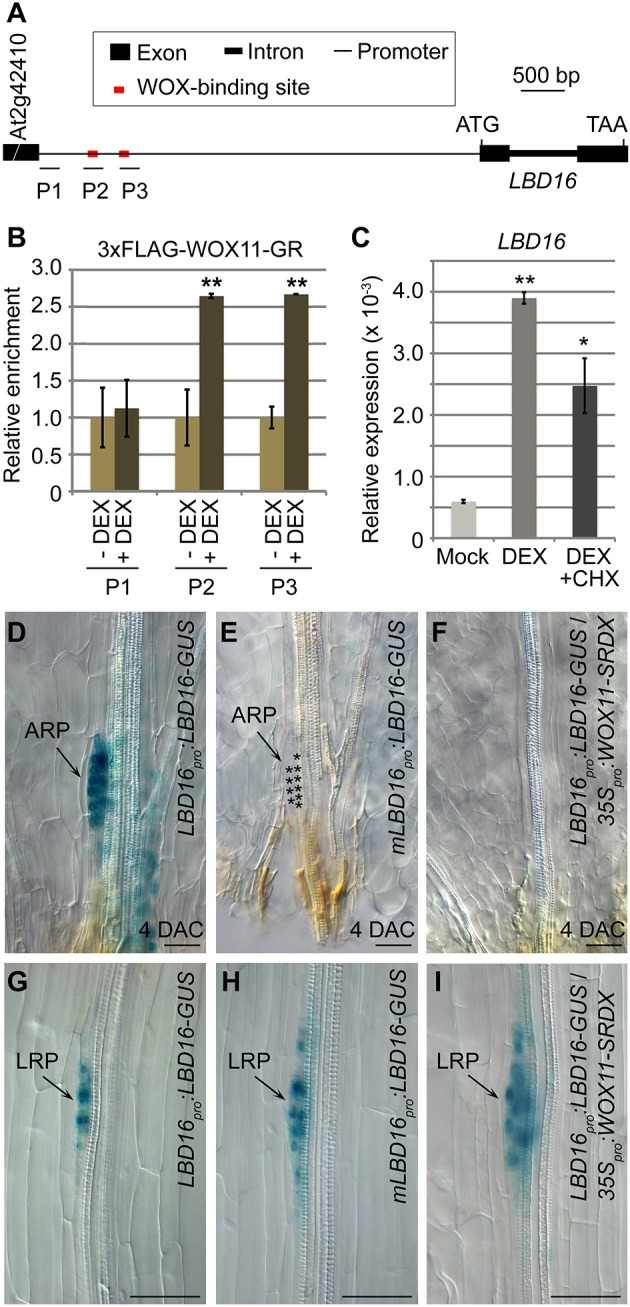
***WOX11* directly upregulates *LBD16* in AR initiation.** (A) Schematic of the structure of the *LBD16* gene. Horizontal lines below the gene show positions of PCR fragments in the ChIP analysis shown in B. (B) ChIP analysis showed that 3×FLAG-WOX11-GR was enriched in the promoter of *LBD16*. Twelve-day-old seedlings grown on 1/2 MS medium were treated with 10 μM DEX (+DEX) or DMSO mock (–DEX) for 4 h. Petioles were harvested for ChIP analysis because petioles contain an abundance of cells competent for adventitious rooting. The ChIP results were normalized to the input control. The values in –DEX were arbitrarily fixed at 1.0. **P<0.01 (two-sample Student's *t*-test compared with –DEX). (C) qRT-PCR analysis of *LBD16* expression in *35S_pro_:3×FLAG-WOX11-GR*. Leaf explants detached from 12-day-old seedlings were treated with DMSO (mock), 10 μM DEX, or both 10 μM DEX and 10 μM CHX on B5 medium for 4 h for RNA extraction. *P<0.05, **P<0.01 (two-sample Student's *t*-test compared with the mock). (D-F) GUS staining of *LBD16_pro_:LBD16-GUS* (D), *mLBD16_pro_:LBD16-GUS* (E) and *LBD16_pro_:LBD16-GUS*/*35S_pro_:WOX11-SRDX* (F) leaf explants cultured on B5 medium at 4 DAC. Leaf explants were detached from 12-day-old seedlings grown on 1/2 MS medium. Asterisks in E indicate ARP cells. (G-I) GUS staining of primary roots from 9-day-old *LBD16_pro_:LBD16-GUS* (G), *mLBD16_pro_:LBD16-GUS* (H) and *LBD16_pro_:LBD16-GUS*/*35S_pro_:WOX11-SRDX* (I) grown vertically on 1/2 MS medium. Error bars represent s.e.m. from three biological repetitions. Each biological repetition was performed with three technical repetitions. Two independent transgenic lines of either *LBD16_pro_:LBD16-GUS* or *mLBD16_pro_:LBD16-GUS* were tested in D-I and the results were consistent. Scale bars: 50 μm.

We first analyzed the promoter sequence of *LBD16* and noted that it contains two WOX-binding *cis* elements (TTAATGG) (Lohmann et al., 2001; Leibfried et al., 2005; Zhao et al., 2009) (Fig. 7A). Results from chromatin immunoprecipitation (ChIP) analysis showed that the 3×FLAG-WOX11-GR protein, in which the WOX11 protein was fused with the 3×FLAG tag and a dexamethasone (DEX)-induced nuclear localization domain GLUCOCORTICOID RECEPTOR (GR) (Aoyama and Chua, 1997), was able to directly bind the promoter of *LBD16* at the predicted binding sites in *35S_pro_:3×FLAG-WOX11-GR* transgenic plants (Fig. 7B).

Upon DEX treatment, *LBD16* expression was upregulated in leaf explants carrying the *3×FLAG-WOX11-GR* construct (Fig. 7C). Moreover, this upregulation of *LBD16* remained when treated with DEX together with cycloheximide (CHX), an inhibitor of protein synthesis, indicating the direct regulation of *LBD16* by WOX11 (Fig. 7C).

To determine the functionality of the identified WOX-binding sites, we compared GUS staining in two reporter lines: one harboring an *LBD16_pro_:LBD16-GUS* fusion and the other carrying the *LBD16* promoter mutated at two WOX-binding sites (*mLBD16_pro_:LBD16-GUS*). GUS staining of *LBD16_pro_:LBD16-GUS* was induced during ARP initiation in leaf explants at 4 DAC (Fig. 7D), whereas GUS staining was barely detected in the *mLBD16_pro_:LBD16-GUS* leaf explants at 4 DAC (Fig. 7E). Moreover, in *LBD16_pro_:LBD16-GUS*/*35S_pro_:WOX11-SRDX* leaf explants, GUS staining was not detected (Fig. 7F). These data suggest that WOX11 acts as a transcriptional activator of *LBD16* mainly through direct binding to the WOX-binding sites during ARP initiation.

On the other hand, during LRP initiation from the primary root grown vertically on medium, the GUS signals of *LBD16_pro_:LBD16-GUS* (Fig. 7G), *mLBD16_pro_:LBD16-GUS* (Fig. 7H) and *LBD16_pro_:LBD16-GUS*/*35S_pro_:WOX11-SRDX* (Fig. 7I) were all clearly elevated. This suggests that WOX11 does not regulate *LBD16* in lateral rooting when plants are grown vertically on 1/2 MS medium.

In summary, our data strongly support the hypothesis that WOX11 directly activates *LBD16* expression in the *WOX11*-mediated rooting pathway. ARF7/19 activate *LBD16* expression in LR initiation in the non-*WOX11*-mediated rooting pathway when plants are grown vertically on medium. Therefore, *WOX11*-mediated and non-*WOX11*-mediated rooting pathways may eventually converge to the activation of *LBD16*.

## DISCUSSION

**Fig. 8.**
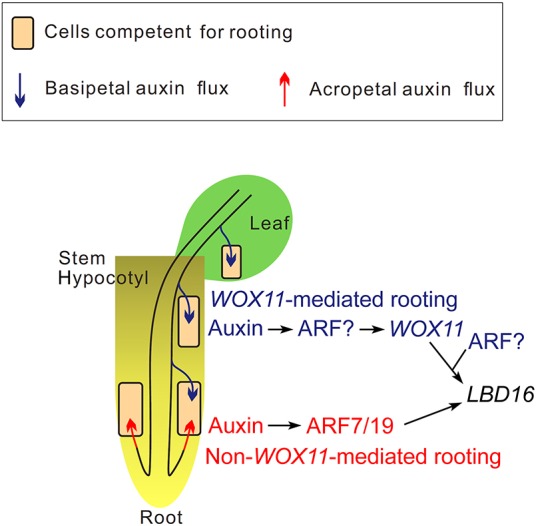
**Model of root system formation in *Arabidopsis*.** *WOX11*-mediated (blue) and non-*WOX11*-mediated (red) rooting pathways contribute to the plasticity of *Arabidopsis* root system formation. *WOX11*-mediated rooting can occur in leaf explants, stem explants and hypocotyls. *WOX11*-mediated rooting can also occur in the root mainly in response to environmental signals, such as wounding or stress. The non-*WOX11*-mediated rooting pathway is dependent on developmental signals to produce LRs. The question marks indicate the proposal that ARF proteins are involved in *WOX11*-mediated rooting.

Based on the present results, we provide a model for the *WOX11*-mediated and non-*WOX11*-mediated rooting pathways in *Arabidopsis* root system formation (Fig. 8).

When plants are vertically grown on 1/2 MS medium, LR formation may heavily depend on developmental signals derived from the root cap (Xuan et al., 2015, 2016). In lateral rooting on medium, the acropetal auxin flux originating from the root cap (Xuan et al., 2015, 2016) probably activates the function of ARF7 and ARF19 in xylem-pole pericycle cells for LR founder cell establishment (Okushima et al., 2007), and this process does not require *WOX11* (non-*WOX11*-mediated rooting in Fig. 8). Wounding or stress could induce AR formation from non-root organs. In adventitious rooting, shoot-derived basipetal auxin flux can accumulate in various types of cells competent for rooting, e.g. procambium and some parenchyma cells in the leaf and stem, and procambium and xylem-pole pericycle cells in the hypocotyl (Sugimoto et al., 2010; Li et al., 2012; Ahkami et al., 2013; Sukumar et al., 2013; Liu et al., 2014; Pacurar et al., 2014). Auxin can then activate *WOX11* for AR founder cell establishment (Liu et al., 2014; Chen et al., 2016; Hu and Xu, 2016) (*WOX11*-mediated rooting in Fig. 8). Both WOX11 in adventitious rooting and ARF7/19 in lateral rooting directly upregulate *LBD16* in founder cells for later primordium development (Fig. 8). Therefore, the distinction between adventitious and lateral rooting appears to be relevant mainly at the initial developmental steps, and *WOX11* is a potential molecular marker to discriminate between the two types of rooting. We hypothesize that some ARF members may directly regulate *WOX11* expression in AR founder cells in response to auxin signaling, as *WOX11* is indicated to be a direct target of the auxin signaling pathway (Liu et al., 2014). Identification of such ARFs will improve our understanding of the missing link from auxin to *WOX11*. We cannot exclude the possibility that *ARF7* and *ARF19* are also involved in some types of AR formation, because a previous study suggested that they are involved in adventitious rooting from hypocotyls (Wilmoth et al., 2005). Currently, it is not clear whether *ARF7*/*19* and *WOX11* are totally independent in all types of AR formation. It is also not clear whether adventitious rooting from leaves, stems or hypocotyls follows exactly the same pathway. Furthermore, we cannot exclude the possibility that other different mechanisms exist in the initiation of root primordium for lateral and adventitious rooting.

In natural conditions, root system formation in soil is complex and is under the control of both development signals and environmental cues such as nutrition, wounding and stress [e.g. root tip wounding, Fig. 3; and drought, Fig. 5 (Verslues et al., 2006)]. In this study, we show that the *Arabidopsis* primary root has the competence to initiate both *WOX11*-mediated roots and non-*WOX11*-mediated roots (i.e. *ARF7*/*19*-mediated LRs) when plants are grown in soil or when the primary root is damaged (Fig. 8). Primary root branching through both *WOX11*-mediated and non-*WOX11*-mediated root formation pathways in soil may represent the co-occurrence of two induction mechanisms that allow integration of developmental signals with environmental cues to optimize plant growth.

We observed that the auxin response is different in the primary roots of plants grown in soil and vertically on 1/2 MS medium, suggesting that different environmental cues may cause different auxin responses in roots (Fig. S12). A higher auxin level in soil (Fig. S12) is likely to lead to higher *WOX11* expression level in soil. Analysis using BAR-Tools (http://www.bar.utoronto.ca/) (Winter et al., 2007; Geng et al., 2013) suggests that NaCl treatment can upregulate *WOX11* expression, indicating that salt conditions may have a role in regulation of root system formation. In this study, the soil condition comprises many nutrients. Nutrients and many environmental signals in soil might also alter the root system (Macgregor et al., 2008; Atkinson et al., 2014; Kellermeier et al., 2014; Otvos and Benkova, 2017). In addition, *LBD16* expression could be induced in the primary root of *arf7-1 arf19-1* in either drought conditions or upon wounding on medium (Fig. S13). The *arf7-1 arf19-1* double mutant and the *WOX11* and *LBD16* marker lines could provide an experimental system for the effects of inductive cues on root branching. Future analysis of the inductive cues will provide a more comprehensive understanding of how root system architecture is established and how developmental plasticity is employed in an effort to optimize response to a variable environment.

## MATERIALS AND METHODS

### Plant materials

The *lbd16-2* (Fan et al., 2012) mutant, the *wox11-2 wox12-1* double mutant (Liu et al., 2014), the *arf7-1 arf19-1* mutant (Okushima et al., 2007), and *WOX11_pro_:GUS* (Liu et al., 2014) and *DR5_pro_:GUS* (Ulmasov et al., 1997) transgenic plants were described previously. For construction of *LBD16_pro_:LBD16-GUS* and *mLBD16_pro_:LBD16-GUS* transgenic plants, the 4.8-kb wild-type or mutated genomic region of *LBD16* was inserted into the pBI101 vector, respectively. *35S_pro_:3×FLAG-WOX11-GR* was constructed by insertion of the cDNA encoding the fused 3×FLAG-WOX11-GR protein into the pMON530 vector. To construct *pER8:3×FLAG-LBD16*, a cDNA encoding the fused 3×FLAG-LBD16 protein was PCR amplified and inserted into the pER8 vector (Zuo et al., 2000). *WOX11_pro_:WOX11* was constructed by insertion of the *WOX11* CDS following the 4.8-kb *WOX11* promoter (Liu et al., 2014) into the modified pBI101 vector in which the *GUS* gene had been removed. *GATA23_pro_:GATA23-GUS* was constructed by insertion of the 1.6-kb genomic region including the promoter and the gene body of *GATA23* into the pBI101 vector. Transgenic plants were obtained by *Agrobacterium tumefaciens*-mediated transformation into wild-type *Arabidopsis* Col-0. Primers used in molecular cloning are listed in Table S1.

### Plant growth conditions

The medium conditions (1/2 MS and B5) were described previously (Chen et al., 2014; Liu et al., 2014). The soil is a 1:1:3 mixture of vermiculite: perlite: peat soil containing 1/2 MS liquid solution without sucrose. Plants were grown in soil in 20-cm-high pots. All plants and explants were grown and cultured at 22°C.

### GUS staining and imaging

GUS staining was carried out based on our protocol (He et al., 2012; Chen et al., 2014). Microscopy and differential interference contrast (DIC) observations were performed as previously described (Chen et al., 2014; Liu et al., 2014) using a Nikon Eclipse 80i microscope.

### ChIP and qRT-PCR

ChIP and qRT-PCR were performed as previously described (He et al., 2012). The qRT-PCR results are presented as relative transcript levels, which were normalized against that of *ACTIN* genes. Anti-FLAG antibody (F1804, Sigma) was used in the ChIP analysis. The primers used for real-time PCR are listed in Table S1.
